# Exploring inequities in access to diabetes technologies among children and young people with type 1 diabetes: Perspectives of parents and young people from ethnic minority groups and low socio‐economic areas

**DOI:** 10.1111/dme.70304

**Published:** 2026-03-29

**Authors:** Eda Tonga, Mark Evans, Nick Oliver, Sze May Ng, Natalie Darko

**Affiliations:** ^1^ Diabetes Research Centre, Division of Global Lifestyle and Metabolic Health, College of Life Sciences University of Leicester Leicester UK; ^2^ Institute of Metabolic Science and Department of Medicine University of Cambridge Cambridge UK; ^3^ Imperial College London UK; ^4^ Faculty of Health, Social Care and Medicine Edge Hill University Ormskirk UK; ^5^ Department of Women's and Children's Health University of Liverpool Liverpool UK; ^6^ Paediatric Department Mersey and West Lancashire Teaching Hospitals Ormskirk UK

**Keywords:** children and young people, diabetes technology, equity, ethnic minority, inequality, qualitative research, type 1 diabetes

## Abstract

**Aims:**

Access to diabetes technology in the UK is significantly influenced by socio‐economic status, ethnicity, and systemic healthcare inequities. This study investigates barriers faced by children and young people (CYP) from ethnic minority backgrounds and/or low socio‐economic areas in accessing diabetes technologies, alongside strategies for equitable improvements.

**Methods:**

Online semi‐structured interviews were conducted across the UK with parents and caregivers of CYP (aged 2–17 years) with type 1 diabetes (T1DM) and young people (aged 14–17 years) from ethnic minority groups and low socio‐economic areas. Participants were recruited via purposive sampling. Interviews were transcribed, coded, and analysed using a thematic reflexive analysis in QSR NVivo12.

**Results:**

Thirty‐two participants were included in the study. Of these, 27 triad interviews were completed involving parents and CYP, along with an additional five triad interviews led by young people. The majority of parents and CYP identified as being from Black African ethnic minority groups (72%), 13% were from Other Black ethnic groups, and a smaller proportion were from Asian minority groups, (8%) and Other ethnic minority groups (6%). Key themes included barriers to accessibility (e.g., financial, linguistic, and geographic challenges), variability in education and service provision, intersectional barriers (e.g., race and socio‐economic status), and the need for improved communication and trust with healthcare professionals. The findings highlight the persistent inequities in diabetes technology access among underserved groups.

**Conclusion:**

Barriers to diabetes technology access for CYP from ethnic minority backgrounds and low socio‐economic areas stem from an interplay of systemic inequities, cultural and linguistic challenges, and financial constraints. This study highlights the need for systemic reforms, including culturally tailored and standardised education programmes alongside equitable resource distribution, to address these barriers.

What's new?

- This study identifies key barriers to diabetes technology access for children and young people (CYP) from ethnic minority backgrounds and low socio‐economic areas, including financial constraints, language and dialect challenges, cultural differences, and systemic healthcare inequities.
- The findings highlight the importance of culturally tailored, standardised education and equitable service provision, alongside improved healthcare professional training to address communication challenges and perceived discrimination.
- This is the first UK study conducted to explore the perceptions of CYP and their parents from ethnic minority backgrounds and/or those living in low socio‐economic conditions regarding barriers to accessing diabetes technology.

## 1 INTRODUCTION

The UK has the highest number of children and young people (CYP) diagnosed with type 1 diabetes (T1DM) in Europe, presenting significant challenges for healthcare services.^1^, ^2^ Continuous glucose monitoring (CGM) and targeted insulin replacement, along with parental engagement, are crucial first steps in managing T1DM in CYP.^3^, ^4^ Advancements in diabetes technology, such as insulin pumps, CGMs and hybrid closed‐loop (HCL) systems, have significantly improved health outcomes by lowering HbA1c levels, reducing the risks of diabetic ketoacidosis and retinopathy, and alleviating stress related to blood glucose management.^5^, ^6^

National organisations, including the American Diabetes Association (ADA), European Association for the Study of Diabetes (EASD), International Society for Paediatric and Adolescent Diabetes (ISPAD) and National Institute for Health and Care Excellence (NICE), recommend the use of these devices.^3^, ^4^, ^7^, ^8^ Most recently, the (2022/2023) National Paediatric Diabetes Audit (NPDA) annual reports published in June 2024 highlighted a growing disparity in access to diabetes technologies among CYP in the United Kingdom (UK), particularly between the most and least deprived areas and between white and ethnic minority groups. It also revealed that individuals using CGM were more likely to achieve lower HbA1c, regardless of the method of insulin delivery.^9^

The National Institute for Health and Care Excellence (NICE2023) Technology Appraisal guidance (TA943) on HCL systems for managing blood glucose in T1DM strongly supports widespread NHS implementation and universal access to these systems for children and adolescents with T1DM.^10^ This represents a significant and positive step toward improving access to advanced diabetes technologies.^9^ Unlike earlier NICE guidelines, which set strict criteria for CGM eligibility, the latest recommendations aim to broaden access, extending it to HCL systems for both children and adults with T1DM.^11^

Despite advancements, access to diabetes care remains shaped by socio‐economic status and ethnicity, with racial and ethnic minority groups experiencing poorer glucose self‐management and persistent gaps in technology access.^12^, ^13^, ^14^ In the UK, NICE guidance exists, yet access remains highly variable due to regional differences in commissioning, resulting in inequities that prevent many from benefiting fully from technologies that could improve diabetes management and quality of life.^14^, ^15^, ^16^

Addressing regional variation is crucial for equitable access to diabetes care. Although studies^17^, ^18^ report disparities in technology use, no UK qualitative studies explore how children, young people, and families experience these inequities.

Intersectionality provides a framework for understanding how multiple social identities and structural forces shape healthcare experiences. Originating from Crenshaw's work on overlapping systems of race and gender (1989, 1991), it has been widely used in public health to explore inequities across multiple axes of identity and power.^19^, ^20^, ^21^ In the UK, this perspective enables examination of how ethnicity, gender, and socio‐economic disadvantage intersect with institutional practices and service delivery to influence access to care.

Although intersectionality has been applied internationally in health research, its use in UK diabetes technology studies is limited. Recent work demonstrates how intersectional analyses can reveal ways technological innovations may inadvertently reproduce inequities, if structural barriers are not addressed.^22^ Informed by this evidence, the present study uses an intersectionality‐informed lens to explore how social and structural factors shape experiences of ethnically diverse and socio‐economically disadvantaged families accessing diabetes technologies. The study aims to fill this gap by investigating the challenges faced by CYP from ethnic minority backgrounds and/or low socio‐economic areas in the UK.^23^

## 2 METHODS

### 2.1 Design

We conducted online, semi‐structured interviews using an interview guide developed through a literature review, public engagement discussion with CYP and discussions among research team members. The semi‐structured interview design ensured that discussions remained focused on the study aims while allowing participants the flexibility to address issues they considered important, including those unforeseen at the outset.

### 2.2 Theoretical and analytical framework

This study was guided by an intersectionality‐informed approach,^19^, ^20^, ^21^ recognising that diabetes technology access is shaped by multiple social identities—such as ethnicity, gender, and socio‐economic background—and the structural, cultural, and policy contexts in which they operate. The study distinguished between single‐axis barriers (e.g., culture, language, or ethnicity alone) and intersecting barriers where multiple social positions co‐occurred (e.g., ethnicity and deprivation; language and geographic access; gender and socio‐economic status).

Our analysis applied an intersectional lens, informed by recent work on diabetes technology inequities,^22^ to interpret how intersecting cultural, digital, and financial factors collectively shaped participants' experiences. This approach provided a theoretically grounded and contextually sensitive understanding of inequities within the UK's publicly funded healthcare system.

### 2.3 Ethics

This study was reviewed and approved by the University of Leicester Research Ethics‐Committee and the Health Research Authority. [REC 23/WS/0095].

### 2.4 Sample and recruitment

We recruited CYP with T1DM, along with their parents or carers, focusing on ethnic minority and/or low socio‐economic groups. CYP were interviewed alongside a family member, guardian, or carer to explore experiences of accessing diabetes technologies and related inequalities. Participants included:

- CYP aged 2–17 years (parents could participate without their child), and
- CYP aged 14–17 years accompanied by a parent, guardian, or carer.

Recruitment used two routes: via NHS Trusts serving ethnically diverse, low‐income communities, and purposive sampling through charities and organisations supporting families affected by T1DM (e.g., JDRF, Diabetes UK, Diabetes Africa, Egality). Participants were drawn from low socio‐economic areas across UK cities including Leicester, Nottingham, Birmingham, and Liverpool, identified using national deprivation indices.

### 2.5 Interviews

Interviews were conducted online via Microsoft Teams, lasting 45–60 min. Sessions were either audio‐recorded and transcribed manually or automatically with Microsoft Teams, with automated transcripts checked for accuracy. Participants were contacted via email. Demographic data collected included parent gender, age, race, education, household income, and child age, race, and year of diagnosis.

The semi‐structured interview guide was developed from literature, clinical experience, and an intersectional framework, then reviewed by clinical advisors and piloted with two parents to ensure clarity, relevance, and cultural appropriateness.

Interviews were conducted by experienced research team members familiar with working with CYP and underserved groups. The team includes diverse races and ethnicities (ND: Mixed Black African; ET: White Turkish) and had no prior clinical relationship with participants. Senior team members include clinicians and clinical academics: MN (Consultant Paediatric Endocrinologist), ME (Clinical Scientist/Consultant in Diabetes and General Medicine), and Professor NO (Clinical Academic and Consultant Diabetologist).

### 2.6 Data analysis

Descriptive statistics were used to analyse and present the demographic characteristics of the study sample, including both parents and their children. The qualitative interview data were organised using QSR NVivo 12 software.^24^ A reflexive thematic analysis was performed following Braun and Clarke's approach.^25^ Two researchers independently coded the transcripts and then compared their coding to identify similarities and discrepancies. Differences were discussed in regular team meetings, and consensus was reached through iterative discussion and refinement of the coding framework until full agreement was achieved.

## 3 RESULTS

Thirty‐two participants took part, including 27 parent–CYP triads and 5 triads led by young people. Findings are reported following the COREQ checklist in Data S2. Interviews were conducted between January and May 2024.

Parents/carers had a mean age of 33.6 years (SD = 7.4, range 21–46), and CYP were aged 14–17. Most participants were from Black African ethnic minority groups (72%), with 13% from Other Black groups, 8% Asian, and 6% Other ethnic minorities. CYP matched their parents' ethnic backgrounds. Most parents had completed high school and were from low‐income households; only 4% earned over £50,000/year.

Participants were geographically diverse: London (42%), East Midlands (15%), Northwest (6%), North East (6%), Yorkshire and the Humber (12%), West Midlands (3%), South East (3%), Brentford (3%), with 9% unspecified. Dropouts tended to have lower education and income. Among CYP, 23% used CGM with insulin pens, while 73% used CGM with insulin pumps or HCL. Participant characteristics are detailed in Table 1.

**TABLE 1:** Characteristics of participants (parents and CYP).

	Mean (SD)
Age parent (years)	33.58 (7.41)
Age child (years)	10.55 (5.84)
Time since diagnosis (years)	4.76 (4.15)

	*n* (%)
Ethnicity	
Black African	23 (71)
Other Black	4 (12.5)
Asian	3 (9.3)
Other ethnic minority group	2 (6.25)
Recruitment	
Cites	4 (12.5)
Social Media	29 (87.5)
Location	
Brentford	1 (3.0)
Coventry	1 (3.0)
Leeds	1 (3.0)
Leicester	3 (9.1)
Liverpool	2 (6.1)
London	14 (42.4)
Newcastle Upon Tyne	2 (6.1)
Nottingham	2 (6.1)
Sheffield	3 (9.1)
Wexham	1 (3.0)
n/a	3 (9.1)
Gender	
Man	23 (71.8)
Women	9 (28.12)
Income	
< 25 k	12 (37.5)
25 k–50 k	12 (37.5)
> 50 k	4 (12.5)
n/a	4 (12.5)
Religion	
Buddhist	1 (3.1)
Christian	16 (50)
Hindu	2 (6.3)
Muslim	13 (40.6)
Education	
GCSE	1 (3.1)
High/Secondary school	9 (27.1)
College	1 (3.1)
Undergraduate	9 (27.1)
Postgraduate	5 (15.6)
n/a	7 (21.9)
Technology Utilisation	
CGM + Insulin pen	8 (25)
CGM + Insulin Pump or HCL	24 (75)

### 3.1 Themes

Five themes, presented below, were identified that illustrate the perspectives of parents and young people in terms of their perceptions and experiences of accessing diabetes technology. Illustrative participant quotes are presented throughout to support each theme and ensure transparency between data and interpretation. Tables 2, 3, 4, 5, 6 illustrate the quotes for each theme. Participants used the following terminology when discussing their technologies: CGM, insulin pump, automated pump or CGM integrated pump, which refers to the HCL.

1. Barriers to Accessibility
2. Perceptions and Experiences of Diabetes Technology
3. Variability in Service Provision and Education for Diabetes Technology
4. Intersecting and Single‐Axis Barriers
5. Enhancing Communication and Trust with Healthcare Professionals for Equitable Access

**TABLE 2:** Barriers to accessibility.

Quote no	Quotes
1	‘And I really wish I could access. I could get those technologies, but… I had a stumbling block which was financial stability’
2	‘Because of the finance issue. But my husband had to run around seeking for assistance from family members’
3	‘We had to …borrow money’
4	‘You need to get the automated pump (HCL), which almost every parent is struggling to get it…it requires a smartphone… but the finances affect a lot of families. It isn't the best’
5	‘But it wasn't widely available to everyone, you had to pay like to get you had to pay to use it, even the Dexcom 7. So, you had to pay’
6	‘Accessing the technologies because you cannot afford what the higher income people can afford… it's something that is very challenging and it gets you really down’
7	‘Young people should have the option to choose the technology that works best for them. It's …hard to cope with diabetes, and they should have the freedom to decide. Right now, some people can't access certain technologies just because their HbA1c levels are better managed, and that doesn't seem fair’
8	‘I only know that when it shows red, when I am called the school team. I become a little bit scared and just pick up the phone and call them and jump in the car’
9	‘Basically, you know all these other nurses and doctors have issue understanding my language…my accent. My accent was the first thing that created a barrier because I'm Caribbean’
10	‘I feel most of these resources should be multilingual, for accents too, [but also] so someone could easily access them without being uncomfortable with English’
11	‘Get some translators more translating, more funding for translators as a whole for the whole language and accent, I guess, healthy communication and culture, but especially diabetes technology, because in ethnic minorities especially, the reason why I think it's slower is the accent barrier’
12	‘The bus journey is about an hour, so if I need to do it so then I'd have to wait till I get to my destination’
13	*‘*My mum and I have to take public transport to the hospital. It's not safe, and the cost is too high. Parking is stressful, and walking isn't always an easy option. When it comes to general appointments, I think there should be more support to help us get to the hospital. For example, my mum wants to take me, but then we think about how much it's going to cost her…the expenses make it hard for her’

#### 3.1.1 Barriers to accessibility

Table 2 presents the quotes for the first theme, *‘Barriers to Accessibility’*, numbered Quote 1–13.

##### Financial barriers

Finance was the most frequently mentioned obstacle to accessing diabetes technologies, with many struggling to afford essential devices. To provide additional context, we have included supplementary data on NHS funding for diabetes technologies. All participants from low‐income groups frequently cited insufficient funding as the primary challenge, particularly when attempting to obtain an HCL or CGM that required a smartphone.

One Black British father of an 11‐year‐old child described the financial burden:

I really wish I could access… those technologies, but I really had a stumbling block …which was financial stability (Quote 1).

Several families reported relying on informal financial support, such as borrowing money or seeking assistance from extended family, to afford essential devices (Quotes 2 and 6). Participants also highlighted the inequity of access, noting that inability to pay excluded them from technologies more readily available to higher income families (Quote 6).

In addition to cost barriers, participants expressed a strong preference for advanced technologies, particularly HCL systems, which they perceived as offering improved glucose management and reduced stress for both children and parents. However, the requirement for smartphones further compounded financial barriers and limited access for many families (Quote 4). Young people also emphasised the importance of having autonomy and choice in selecting technologies that best supported their daily diabetes management (Quote 7).

Parents described heightened anxiety when managing glucose fluctuations without access to advanced technologies (smartphone remote monitoring), particularly during school hours, which increased fear and reliance on emergency responses rather than preventative management (Quote 8).

###### Linguistic barriers: Accent and dialect challenges

Participants identified communication difficulties related to accent and dialect as a significant barrier to accessing diabetes care. Participants emphasised the need for more inclusive, multilingual, and accent‐sensitive resources to reduce discomfort and improve communication (Quotes 9 and 10).

One Black African parent with a 4‐year‐old child described this challenge:

Basically, all the… nurses and doctors have issues understanding my…my accent… my accent was the first thing that created a barrier (Quote 9).

###### Geographic challenges and related costs

Families living in remote or underserved areas described distance to diabetes clinics as a major barrier, compounded by long travel times, reliance on public transport, and associated costs. Participants highlighted the physical, financial, and emotional burden of attending appointments, particularly for families already facing economic constraints (Quotes 12 and 13). One participant explained the impact of travel time:

The bus journey is about an hour, so if I need to do it… I'd have to wait quite a while till I get to my destination (Quote 12).

#### 3.1.2 Perceptions and experiences of diabetes technology

Table 3 presents supporting quotations for this theme (Quotes 1–12). Overall, participants expressed positive perceptions of diabetes technologies, describing improvements in diabetes management, quality of life, and confidence in self‐care. Many highlighted the transformative role of CGM, pumps, and particularly HCL systems in supporting daily management (Quotes 2 and 3).

**TABLE 3:** Perceptions and experiences of diabetes technology.

Quote no	Quotes
1	‘I think the diabetes technology can improve the management of type of diabetes’
2	‘Without it, I feel managing my diabetes would have been extremely difficult’
3	‘I could also say that I see promising results in the future if there are proper management and umm improvements and more information to better this product…the tech industry leaflets are not great’
4	‘What I'm going for is like ….[that] it's reliable’
5	‘I found that one, the automated pump (HCL), to be more comfortable… because myself even I am an adult. I still cling away from my needles so it's… automated is better and because if there is an emergency, I may not be able to do anything as a parent’
6	‘I think it's amazing cause of how fast they've managed to like the progress is amazing…but since the development of glucose monitors and pumps …my diabetes management's a lot better and my HBA1C is better as well’. (Young person)
7	‘Yeah, there were actually helpful because even umm, before learning from my doctors, I did watch some videos and you know those videos [on You Tube] were actually by health professionals’
8	‘I can't even Google it. I can only go to the hospital after the first mistake. I almost lost my daughter because of that. I can never access it. I only get my information from the hospital directly’
9	‘Yeah, about a personal health information that is being stored in the device. Yeah, it's…something that is also worrisome’
10	‘I have the insulin pump, which has been really helpful and the continuous glucose monitoring and that has really been helpful for me and it's easy to manage because these technologies are linked to devices which it's easy to monitor and keep track of anything you know’
11	‘But at first it might be a lot more difficult for you to be able to navigate that because you're not in the field or you're having to make use of that before. So, it might give you some little tough time, but it just depends on how committed you are to learning it. So, at first it is difficult… although they give you some directives, some manual on things to do’
12	‘I got it from the hospital. But I think I learned how to use it mainly from the Internet because I don't know if I could understand’

One Black African Caribbean male young person (17 years) explained:

Without the technology, I feel managing my diabetes would have been extremely difficult (Quote 2).

Participants were also optimistic about future developments in diabetes technologies, particularly HCL systems, while noting the need for better information and clearer guidance to maximise their benefits (Quote 3).

##### Reliability and ease of use

Reliability was a key factor influencing technology choice, with participants placing high value on recommendations from diabetes consultants and GPs when selecting devices. Ease of use and automation, particularly in emergency situations, were viewed as critical advantages of HCL systems, especially for parents managing young children's diabetes (Quotes 4 and 5). Participants also noted that technological advancements had significantly improved glycaemic control and overall ease of management over time (Quote 6).

One parent described their preference for automated systems:

I found that one, the automated pump [HCL], to be more comfortable… because it's automated (Quote 5).

##### Learning and accessibility of resources

Most participants reported learning to use diabetes technologies through a combination of healthcare professional support and self‐directed resources, such as online videos (You tube videos with healthcare professionals), and brochures that they had located themselves (Quote 7). However, some described initial difficulties navigating devices and highlighted the need for clearer, more accessible, and multilingual guidance to better support diverse users (Quote 8, 11, 12).

##### Trust in data accuracy and digital applications

Participants expressed mixed levels of trust in digital applications. Some relied exclusively on healthcare professionals for information, expressing scepticism about online sources (Quote 8), while others raised concerns about the safety and storage of personal health data within devices (Quote 9).

#### 3.1.3 Variability in service provision, training and education for diabetes technology

Table 4 presents supporting quotations for this theme (Quotes 1–11). Participants reported substantial variability in the quality and provision of diabetes technology education across NHS trusts and centres. While most received some form of training from healthcare professionals, the content, depth, and delivery varied considerably between hospitals, shaping confidence and self‐management outcomes (Quotes 2 and 3).

**TABLE 4:** Variability in service provision and education for diabetes technology.

Quote no	Quotes
1	‘I received some, some, some sort of management educations on that, but not really in depths’
2	‘But when I came to Sheffield, it has a Children's Hospital. So the set up was 10 times better …very much more organised. So I think it varies team to team, but my team now is like very good’
3	‘They conducted a long session with three people in the room, and all the doctors were helping throughout. They provided… the actual pump itself and directed us to resources like websites and videos on YouTube. There was so much available that you can use to understand your pump and learn how to use it effectively’ (Young person)
4	‘Have this kind of knowledge when in the learning process is very slow, so when things are like consistently or continually reviewed, it would help. So, better orientation is one of the recommendations’
5	‘I have to go online to Google it, then I can go step by step when I see it online. And that has really been helpful’
6	‘Oh yeah, and also like I would say, practical examples. Where that don't physically or let me in the social media anyhow. But on practical example, if that's. I read ohh different articles on websites social media that really helps’
7	‘I just send in random questions on e‐mail to the consultant… that helps me a lot to be sure about things… So it's a kind of close bond that allows me to access her, and to be able to ask questions. That helped me a lot to access the tech and to be sure about using it. I wouldn't have access without her …[funding for the tech]’
8	‘We can just call anytime in times of emergency; we have all the information we need. They tell us …what can you do and what you need to do…when you need someone to talk to. This is what is happening, and the person is an expert on the other end’
9	‘I would say the educational issue from them was, is, is very helpful even up to now because they make sure that I learnt everything step by step and everything was how to manage and everything was open to me broadly so I would know every single step to manage everything that happens’
10	‘There should be more centralized education from the NHS… The health professionals should also train in terms of understanding the stress, stigma, and behaviour of minority young people to get useful and relevant information for them’
11	‘Our access to technology and support often depends on the individual healthcare professionals—whether they are well‐trained, supportive, and actually there for us’

One participant highlighted differences between services:

When I came to Sheffield, …the setup was 10 times better… It was very much more organised (Quote 2).

Group‐based education sessions were viewed positively, particularly when they enabled interaction with multiple healthcare professionals and access to practical resources such as videos and online materials (Quote 3). In contrast, participants attending centres with limited training provision often relied on self‐directed learning through online sources or social media to compensate for gaps in formal education (Quote 5).

### 3.2 A call for standardised and inclusive education

Participants strongly advocated for standardised, centrally coordinated diabetes technology education across the UK to ensure equitable access to consistent, high‐quality training for all children, young people, and families. They emphasised that education should also address psychosocial factors, including stress and stigma, particularly for ethnic minority CYP (Quote 10). One participant explained:

There should be more centralised education from the NHS… [with] understanding the stress, stigma, and behaviour of minority young people (Quote 10).

Culturally and linguistically sensitive training was identified as essential for effective learning, with participants noting that supportive, well‐trained healthcare professionals fostered greater trust and engagement (Quote 11). Participants also highlighted the importance of ongoing, nationally coordinated education and regular review to reinforce learning and build confidence in diabetes technology use over time (Quote 4).

#### 3.2.1 Intersecting and single‐Axis barriers

Table 5 presents supporting quotations for this theme (Quotes 1–12). Participants described both single‐axis barriers (e.g., language, culture, or ethnicity alone) and intersecting barriers arising from the interaction of multiple social and structural factors. Guided by the intersectionality‐informed framework outlined in the Methods, we distinguished between these two forms of barriers to avoid overextension of the term ‘intersectional’, while capturing where overlapping determinants produced distinctive inequities in access and engagement. For example, Quotes 8, 9, and 11 illustrate how language, culture, race, ethnicity, and socio‐economic status intersected to influence experiences of access, communication, and affordability of diabetes technologies.

**TABLE 5:** Intersecting and single‐axis barriers.

Quote no	Quotes
1	‘If you're talking about inequality, it means maybe they're not giving equal services to both minority people like us, and majority people’
2	‘Umm, so aside from individual financial status, I yeah, I think sometimes that black people always have issues with you know accessing healthcare in general sometimes because due to discrimination’
3	‘Necessary. So, it's a very big factor, but a high income can mostly afford it or easily afford it. So, it's not a factor for them or it's not an issue for them, but it is an issue for me’
4	‘With regards to technology, I don't have the luxury to jump on any new technology… let me call it ‘demographical’ barriers’
5	‘I feel my race … played a very big role in how I was trying to access services’
6	‘From my experience… practitioners from a different race as a patient… tend to support those of the same race more often’
7	‘It would help to make this knowledge for everybody like us…because some people [like us] don't have the knowledge… most people don't’
8	‘Currently, it is my language and my colour… and a little bit of discrimination is involved’
9	‘You need to have resources for the people according to the languages according to the ethnic minority new settlers in our area, in, in and their population’
	‘For instance, we are mostly Islamic in our area, so you need someone who understands Arabic and the culture to reach out’
10	‘I think a little bit of training and orientation for the nurses. I think nurses, should get access to an upgrading of the way that they are being taught’. ‘There should also be more types of training for nurses to help them deal with patients who need help in different ways… languages… our culture’
11	‘The NHS needs to make these technologies accessible… because [like me] some other parents might not have the money I have right now’
12	‘It doesn't seem fair. If you're managing your diabetes well, you still can't get it. Some people in the same (ethnic) group as me, don't know what to say, and their parents are confused too. Healthcare professionals should go to local places and help people understand what they need to do to get access’

##### Race, socio‐economic status, and perceived discrimination

Participants from ethnic minority backgrounds frequently reported feeling disadvantaged when attempting to access diabetes technologies, particularly in relation to affordability and perceived inequitable treatment within healthcare settings (Quotes 4 and 6).

One Black African Caribbean father of a 6‐year‐old child explained:

With regards to technology, I don't have the luxury to jump on any new technology… let me call it “demographical” barriers (Quote 4).

##### Knowledge gaps, language, and cultural differences

Participants highlighted limited awareness of diabetes technologies within some ethnic minority groups, alongside language, accent, and cultural differences that contributed to exclusion and perceived discrimination (Quotes 7 and 8). They emphasised the need for culturally competent and linguistically inclusive care.

One Black African Caribbean parent described this intersection of barriers:

Currently, it is my language and my colour… and a little bit of discrimination is involved (Quote 8).

Participants also stressed the importance of healthcare professionals receiving training that reflects cultural, linguistic, and faith‐based diversity to improve communication and engagement (Quotes 9 and 10).

##### Intersectional inequities in healthcare access

Many participants described how race, ethnicity, socio‐economic status, and cultural context interacted to intensify barriers to diabetes technology access, creating inequities that could not be explained by single factors alone (Quote 11).

One participant noted:

The NHS needs to make these technologies accessible… because some other parents might not have the money I have right now (Quote 11).

Young people also observed inequities within their communities, highlighting how peers who managed their diabetes well were still unable to access technologies due to structural and informational barriers (Quote 12). Participants called for more proactive, community‐based engagement by healthcare professionals to support equitable access.

#### 3.2.2 Enhancing communication and trust with healthcare professionals: Educational recommendations

Table 6 presents supporting quotations for this theme (Quotes 1–15). Participants consistently described variability in the attitudes, communication styles, and competencies of healthcare professionals, which influenced their care experiences. Inconsistent training and limited understanding of the needs of ethnically and culturally diverse families were perceived to undermine trust and engagement and feelings of being treated impartially (Quotes 2 and 3).

**TABLE 6:** Improving communication with healthcare professionals and recommendations.

Quote no	Quotes
1	‘Have really put in people of ethnic minorities, but if I were to speak. Of a way, if we could be included because I feel this an exclusion right now, but if we should be more included in healthcare, it must be with someone you trust, someone that understands your background. Because if I'm with a doctor, a doctor, that that is from my own ethnicity with me … I'll be able to trust more’
2	‘So really, I would say it's just it depends on the individual healthcare professionals. Some are not well‐trained, and they're not friendly at all’
3	‘They should do … better, never be biased, and to help us access as much help as they can’
4	‘Friendly impressions because my like I said before, my accent seem difficult for them, so they don't understand me and are not very patient. Always make sure that we understand everything to the end. Make sure they're very patient and friendly’
5	‘I think government should subsidise the cost of the support therapy’
6	‘We are not asking for grants, but they could come to our aid… for us to have a culture of checking ourselves and checking our children by making available funds for intervention and lowering the cost of technologies [we can access] and services’
7	‘So, when things are like consistently or continually reviewed …, it would help’
8	‘Education, education have been open seminars, orientations and conference meetings for the people in your in your environment, in your settlement, having a lot of orientations, yes, having conference meetings’
9	‘For instance, we are mostly Islamic in our area, so you need someone who understands Arabic and the religion…culture to be able to reach out’
10	‘Seminars and programmes that educate, you know, the NHS officials and then also. More education. And then there should be laws or policy to sanction this’
11	‘People like on the healthcare professionals, the energy should try your possible best in making sure that everyone perspective of ones on Rachel or ethnic backgrounds have equal to receiving services because there are lots of people out there who are unwilling to go and seek for services’
12	‘Professionally, I would recommend the visuals working with kids and easy‐read as possible’
13	‘Organizing indoor workshops for kids with diabetes to… kind of get in contact and bond with kids with similar experiences. Also…visuals and animations could help kids understand better, especially for those who struggle with traditional leaflets and methods’
14	‘There you could try to reach out to religious organizations must, especially their leaders’
15	‘The best way would be to send in person representatives from different community, poorer areas. Ethnic minority ambassadors to try and reach out to young people’

One Black Caribbean young person explained:

It depends on the individual healthcare professionals. Some are not well‐trained, and they're not friendly at all (Quote 2).

Participants emphasised the importance of empathy, impartiality, and culturally sensitive care, recommending a more standardised approach to professional training to reduce inequities. Equitable access to diabetes technologies and associated training was viewed as essential, with participants highlighting the need for financial support and cost reduction to enable families to engage effectively in diabetes management (Quote 6).

One Black African parent noted:

We are not asking for grants, but they could come to our aid… by making available funds for intervention and lowering the cost of technologies (Quote 6).

Building on concerns about professional training, participants also highlighted the need for tailored educational resources to support learning and confidence, and sustained engagement. Participants further emphasised the importance of community‐based outreach delivered through trusted intermediaries, including faith leaders and ethnic minority community ambassadors, to engage young people in underserved areas (Quotes 14 and 15).

In‐person workshops, peer interaction, and visual learning tools were viewed as particularly beneficial for CYP with T1DM, especially those who struggle with traditional written materials (Quote 13).

One female Black Caribbean young person suggested:

Organising indoor workshops for kids with diabetes… visuals and animations could help kids understand better (Quote 13).

## 4 DISCUSSION

This study provides critical insights into barriers faced by CYP with T1DM diabetes from ethnic minority and low‐income backgrounds in accessing diabetes technologies. Participants emphasised the transformative role of technologies such as CGM, insulin pumps, and HCL systems in simplifying management and improving quality of life, yet barriers persist, rooted in financial, linguistic, cultural, and systemic inequities.

Financial and systemic barriers were prominent. Families from low‐income groups faced difficulties accessing advanced technologies, many of which require smartphones for optimal use, yet smartphones are not routinely funded by the NHS.^12^, ^25^, ^26^ National audit data show persistent disparities: 48.6% of Black CYP use real‐time CGMs versus 62.6% of White peers,^9^, ^15^ reflecting broader geographic and resource inequalities within the NHS.^13^, ^14^, ^16^ Addressing these gaps requires policy‐level reforms and standardised service delivery.

Intersectional challenges were frequently reported, including compounded effects of financial hardship, language barriers, cultural differences, and systemic bias. Participants highlighted how accents, dialects, and cultural misunderstandings undermined communication and trust with healthcare providers, consistent with prior work on language and cultural barriers.^17^, ^18^, ^27^, ^28^ Tailored resources, including multilingual materials and culturally competent care, were deemed critical for improving engagement.

Variability in diabetes education and support was also highlighted. Some hospitals offered comprehensive group training, while others provided minimal instruction, with those accessing high‐quality education reporting better outcomes.^29^ Many relied on self‐directed online learning, which was often insufficient.^30^ Consistent, tailored, face‐to‐face education was recommended to improve self‐efficacy and outcomes.

Healthcare professionals' attitudes and competencies were central to experiences. Participants called for enhanced training in cultural competence, empathy, and communication, aligning with person‐centred care recommendations to reduce health inequities.^16^, ^28^, ^31^, ^32^, ^33^ Training tailored to diverse populations, including remote services, is crucial to improve both communication and equitable care delivery.

This study demonstrates the relevance of intersectionality in understanding access barriers. Race, socio‐economic status, and cultural background interacted in participants' accounts to produce compounded challenges, requiring holistic, multifaceted solutions. The findings align with complementary research exploring healthcare professionals' perspectives, highlighting systemic and interpersonal challenges, including unconscious bias, regional disparities, and the need for culturally competent training.^33^

Applying an intersectionality‐informed framework allowed situating participants' narratives within structural and cultural contexts. Many accounts reflected intersecting identities such as ethnicity and deprivation, or language and gendered caregiving roles producing compounded disadvantage. These findings extend previous applications of intersectionality in diabetes research^22^ and earlier theoretical work by Crenshaw (1989, 1991) and Bowleg (2012) to a UK context.^19^, ^21^ They demonstrate how intersecting social determinants shape opportunities to benefit from healthcare innovation within a publicly funded system, highlighting the need to address not only clinical and economic barriers but also their interaction to achieve equitable diabetes technology access.

### 4.1 Strengths, limitations, and future directions

This study provides important insights into barriers faced by CYP from ethnic minority and low‐income backgrounds in accessing diabetes technologies. A key strength is the inclusion of both CYP and their parents, enabling a holistic understanding of barriers at individual and family levels. By focusing on underrepresented groups, the study addresses a critical evidence gap in UK diabetes research. The qualitative design, using semi‐structured interviews and reflexive thematic analysis, allowed in‐depth exploration of lived experiences and intersectional barriers shaped by socio‐cultural and systemic influences.

Several limitations should be acknowledged. Digital exclusion, including poor connectivity, lack of devices, and limited digital literacy, contributed to participant dropout. Future studies should consider alternative engagement methods, such as in‐person or community‐based approaches, to better include digitally disadvantaged families. Regional variation in diabetes technology access and commissioning may also have influenced perceptions of inequity, highlighting the need for longitudinal or mixed‐methods research to examine how NHS commissioning practices shape access across regions.

Despite purposive recruitment across geographically and socio‐economically diverse UK settings, no White participants were recruited. This reflects both the demographic composition of targeted areas and the study's intentional focus on groups most affected by inequities in diabetes technology access. While this limits generalisability and precludes direct ethnic group comparisons, the findings remain highly relevant and transferable to populations experiencing similar structural disadvantages, particularly in relation to affordability, health system navigation, and culturally appropriate support. Future research could broaden recruitment to include White participants from diverse socio‐economic backgrounds to explore how intersecting social determinants shape access across ethnic groups.

This analysis prioritised the lived experiences of CYP and parents, focusing on individual and interpersonal barriers rather than detailed examination of structural mechanisms. Ongoing work extends these findings by examining institutional, socio‐economic, and policy‐level drivers through the inclusion of healthcare professionals' perspectives.^33^ Future research will build on this by co‐designing family‐informed recommendations, developing culturally and linguistically tailored resources, and generating policy guidance to support equitable implementation. This will include analysis of NHS commissioning, clinic capacity, and funding mechanisms to inform sustainable, inclusive models of diabetes technology provision across the UK.

## 5 CONCLUSION

This study highlights intersecting inequities in access to diabetes technologies for CYP from ethnic minority and low‐income backgrounds. Financial, cultural, linguistic, and geographic barriers, compounded by systemic healthcare biases, limit access to CGM, insulin pumps, and HCL systems. Equitable diabetes care requires standardised education alongside culturally and linguistically tailored support, including multilingual resources, accent‐informed materials, and community‐based interventions. Co‐design with children, young people, and families is essential to ensure interventions are relevant, accessible, and effective. National recommendations could improve free access and embed systemic reforms within policy and practice. Prioritising the voices of ethnic minority and low‐income families is critical to developing scalable, meaningful solutions that advance equitable diabetes care.

## AUTHOR CONTRIBUTIONS

N.D led the qualitative study, designed this interview study with CYP and input from S.M.N, who conceived and designed the wider UNBIASED study. E.T and N.D. collected the data, which was then analysed by E.T, and N.D and an independent qualitative reviewer. ET and ND drafted the manuscript with input from S.M.N. All authors reviewed, edited and approved the final version.

## FUNDING INFORMATION

This is funded by Diabetes UK (Grant reference number: 22/0006434) with Professor Sze May Ng OBE (Chief Investigator), Associate Professor Natalie Darko. Professor Mark Evans, Professor Julia Lawton, and Professor Nick Oliver as co‐investigators. Dr. Eda Tonga supported Unbiased project by Diabetes UK. The views expressed are those of the author(s) and not necessarily those of the funding body. This study has been delivered through the National Institute for Health and Care Research (NIHR) Leicester Biomedical Research Centre. The University of Cambridge has received salary support for MLE from the National Health Service in the East of England through the Clinical Academic Reserve. The views expressed are those of the author(s) and not necessarily those of the NIHR Leicester BRC, the NIHR or the Department of Health and Social Care.

## CONFLICT OF INTEREST STATEMENT

S.M.N. declares honorarium from Sanofi and Insulet. M.E. has been a member of advisory panels and/or received speakers fees from NovoNordisk, Eli Lilly, Abbott Diabetes Care, Medtronic, Dexcom, Ypsomed, Pila Pharma, and Zucara. N.O. reports grants paid to their institution from National Institute for Health and Care Research, Diabetes UK, Helmsley Trust, Dexcom and Medtronic Diabetes; speaker fees from Tandem Diabetes, Sanofi, Dexcom, Astra Zeneca and Medtronic Diabetes; and participation on advisory board for Medtronic Diabetes and Roche Diabetes. ET, ND, MN, have no conflict of interests to declare.

## Supporting information


**Data S1** Diabetes Technologies and NHS Funding Provision Status (2024–25).

**Data S2** COREQ (COnsolidated criteria for REporting Qualitative research) Checklist.

## Data Availability

The datasets generated and analysed for this study are not publicly available due to risks to individual privacy. However, the quote tables have been provided and the data sets are available, via the corresponding author, on reasonable request.
