# Supplementary material for: Exploring inequities in access to diabetes technologies among children and young people with type 1 diabetes: Perspectives of parents and young people from ethnic minority groups and low socio‐economic areas
Source: Diabet Med. 2026 Mar 29;43(7):e70304. doi: 10.1111/dme.70304 (PMC13257907; doi:10.1111/dme.70304)
Supplement: Supplementary file 1 — Data S1 Diabetes Technologies and NHS Funding Provision Status (2024–25). [file DME-43-e70304-s002.docx]

**Supplementary data:** Diabetes Technologies and NHS Funding Provision Status (2024-25)

| **Diabetes Technologies** | **NHS Funding Status** |
| --- | --- |
| **Blood Glucose Monitoring Devices** |  |
| Finger-Prick Meters | **Funded by NHS** |
| Continuous Glucose Monitors (CGMs, e.g., Dexcom G6) | **Funded by NHS** (if clinical need is met under NICE guidelines) |
| Flash Glucose Monitors (e.g., FreeStyle Libre) | **Funded by NHS** (if clinical need is met under NICE guidelines) |
| Dexcom G7 (Advanced CGM) | **Not routinely funded by NHS** |
| Eversense Implantable CGM | **Not funded by NHS** |
|  |  |
| **Insulin Delivery Systems** |  |
| Insulin Pens and Smart Insulin Pens (with tracking features) | **Funded by NHS** |
| Insulin Pumps | **Funded by NHS** (if clinical criteria are met under NICE guidelines) |
| Tubeless Insulin Pumps (e.g., Omnipod DASH) | **Not routinely funded by NHS** |
| Advanced Dual-Hormone Pumps (insulin and glucagon) | **Not funded by NHS** |
|  |  |
| **Hybrid Closed-Loop Systems** |  |
| Hybrid Closed-Loop Systems (e.g., Medtronic MiniMed 780G) | **Funded by NHS** (availability varies by region; NICE guidelines being implemented) |
| Omnipod 5 | **Not routinely funded by NHS** |
|  |  |
| **Supportive Apps and Software** |  |
| DigiBete App | **Free and funded by NHS** |
| mylife App | **Free and funded by NHS** |
| Advanced CGM/Insulin Pump App Features | **Some features require private subscription** |
|  |  |
| **Accessories** |  |
| Smartwatches for CGM Integration | **Not funded by NHS** |
| Insulin Pen Caps with Smart Tracking (e.g., Timesulin) | **Not funded by NHS** |
